# Pre-exercise mood, sex-specific hormonal and neuromodulatory responses to maximal exertion in elite rowers

**DOI:** 10.3389/fphys.2026.1845286

**Published:** 2026-06-12

**Authors:** Joanna Ostapiuk-Karolczuk, Anna Kasperska, Hanna Dziewiecka, Justyna Cichoń-Woźniak, Wojciech Gruszka, Piotr Basta, Sabina Kaczmarczyk, Małgorzata Reysner, Anna Skarpańska-Stejnborn

**Affiliations:** 1 Department of Biological Sciences, Poznan University of Physical Education, Faculty of Sport Sciences, Gorzów Wielkopolski, Poland; 2 Department of Physical Education and Sports, Poznan University of Physical Education, Faculty of Sport Sciences, Gorzów Wielkopolski, Poland; 3 Department of Palliative Medicine, University of Medical Sciences, Poznan, Poland

**Keywords:** cortisol, fatigue, mood state, neurotransmitters, POMS, serotonin-dopamine balance, sex differences

## Abstract

Acute hormonal and neuromodulatory responses to maximal exertion are key indicators of physiological stress in elite athletes, yet it is unclear whether these responses vary with pre-exercise psychological state or differ by sex. This study examined sex-specific links between pre-exercise mood and acute physiological reactions. Twelve elite female (18.75 ± 0.75years) and sixteen elite male (19.43 ± 1.09 years) rowers performed a maximal-effort 2000 m test. Pre-exercise mood was assessed using the Profile of Mood States (POMS). Venous blood collected at PRE, POST, and 3-hour recovery (3H REC) was analyzed for cortisol, testosterone, serotonin, dopamine, gamma-aminobutyric acid (GABA), and tryptophan. Two-way ANOVA and exploratory correlations evaluated sex-specific physiological and psychophysiological responses. Cortisol showed a significant Time × Group interaction (*p* = 0.0002), with males displaying increases at POST and 3H REC, while females showed only small, non-significant changes. Dopamine also exhibited a significant interaction (*p* = 0.0107): males showed PRE-POST elevations and declines during recovery, whereas females showed minimal fluctuations. Tryptophan demonstrated main effects of Time (*p* = 0.0437) and Group (*p* < 0.0001), with larger decreases in females. Psychophysiological associations were sex-specific. In females, negative mood dimensions correlated with lower cortisol at 3H REC (*r* = -0.63 to -0.66). In males, lower vigor and higher depression correlated with higher serotonin and lower dopamine POST (*r* = -0.52 to -0.56). Pre-exercise psychological state was associated with distinct acute hormonal and neuromodulatory responses to maximal exertion, with clear sex-specific patterns. Incorporating mood assessment alongside biochemical markers may support more individualized interpretation of fatigue and recovery markers in elite sport.

**Trial registration:**

This observational study was retrospectively registered atClinicalTrials.gov (NCT07243613).

## Introduction

Elite athletes frequently undertake periods of intensified training designed to provoke physiological adaptation through repeated high-intensity sessions and limited recovery opportunities. These microcycles create a sustained physiological load that accumulates over time, making athletes more responsive to the stress of additional maximal-effort tests. As noted by Meeusen et al. (2013), the combined influence of training load and acute exercise provides an ecologically valid context for examining psychophysiological mechanisms underlying fatigue and recovery, including psychological and cognitive responses associated with accumulated training stress (Valdesalici et al., 2026).

Insufficient recovery during intensified training can lead to transient disturbances across multiple physiological systems. These include elevated cortisol secretion, an imbalance between anabolism and catabolism, increased muscle-damage biomarkers, and activation of inflammatory pathways (Halson, 2014). Such responses often precede measurable decrements in performance and are therefore considered early indicators elevated physiological strain and insufficient recovery. Studying acute responses in this context allows for a more comprehensive understanding of how athletes adapt, or show physiological and psychological responses to accumulated physical and emotional stressors. These multidimensional responses are increasingly recognized as important components of athlete monitoring in high-performance sport (Tian et al., 2026). Mood state is one of the most sensitive indicators of training-related stress. Prolonged or intensified training consistently produces increases in fatigue, tension, and confusion, accompanied by reductions in vigor and motivation (Morgan et al., 1987; Bonin et al., 2025). These shifts frequently emerge before objective performance declines and are commonly detected through routine monitoring. The Profile of Mood States (POMS), in particular, provides a validated framework for detecting early changes in psychological state associated with elevated training stress and recovery demands. Beyond its role in tracking athlete readiness, the POMS has also been associated with performance outcomes. A meta-analysis of 25 studies demonstrated that pre-competition Total Mood Disturbance (TMD) reliably forecasts competitive performance across sports (Lochbaum et al., 2021). Therefore, psychological state may be considered both a marker of accumulated training stress and a potential correlate of subsequent physiological stress reactivity.Cortisol and testosterone provide key endocrine indicators of the body’s response to intense exercise. Cortisol rises in response to metabolic or psychological stress via the activation of the hypothalamic-pituitary-adrenal (HPA) axis, while testosterone supports anabolic recovery and tissue repair through the hypothalamic-pituitary-gonadal (HPG) axis. Their testosterone to cortisol ratio (T/C) is commonly interpreted as an index of anabolic-catabolic balance. Intensified training often elevates cortisol while leaving testosterone unchanged or slightly reduced, resulting in transient reductions in T/C that signal elevated physiological strain (Rowell et al., 2018; Ficarra et al., 2024; Aydemir et al., 2025). Similar endocrine responses have been observed in athletes exposed to intensified training camps or competitive tournament loads, reflecting the cumulative physiological demands associated with repeated exercise exposure (Ostapiuk-Karolczuk et al., 2024; Pancar et al., 2026). These hormonal changes are integral to understanding the recovery process, as they influence energy mobilization, immune function, and tissue remodeling.

Acute intense exercise also affects circulating neurotransmitter-related markers that respond dynamically to exercise stress and may provide additional information about psychophysiological responses associated with fatigue and recovery. A widely accepted model suggests that the balance between serotonergic and dopaminergic activity shapes motivation, arousal, and effort regulation (Meeusen, 2006). Increased serotonergic activity has been associated with lethargy and heightened perceived exertion, while dopaminergic signaling supports alertness and motor drive. In parallel, circulating serotonin and dopamine, derived largely from peripheral sources including platelets, the gastrointestinal tract, adrenal tissue, sympathetic nerve terminals, and immune cells (Eisenhofer et al., 2004; Berger et al., 2009), are increasingly examined in exercise physiology as serum neurochemical markers of systemic responses to acute exercise stress. Although still relatively rarely investigated in elite sport settings, these peripheral markers may provide valuable complementary information for psychophysiological monitoring of fatigue and recovery in athletes (Ostapiuk-Karolczuk et al., 2025b).

Studies consistently report elevated circulating serotonin after strenuous exercise, typically accompanied by increased fatigue perception (Heijnen et al., 2016; Zimmer et al., 2016; Ostapiuk-Karolczuk et al., 2025b). Dopamine generally rises due to sympathetic activation, though the magnitude and timing vary across exercise modalities (Meeusen, 2006). When serotonin rises more than dopamine, it may indicate a shift toward monoamine-related fatigue processes. Emerging evidence suggests that γ-aminobutyric acid (GABA) may also respond to exercise and recovery interventions, potentially reflecting muscle-to-brain signaling pathways (Lyssikatos et al., 2023a; Yang et al., 2024). Although current knowledge about GABA responses to exercise is limited, available evidence suggests that GABA may clarify neurochemical responses associated with fatigue and post-exercise recovery.

In addition to these neurochemical mechanisms, biological sex may further shape physiological and psychological responses to exertion. Some studies indicate that female athletes may show more pronounced mood disturbances or reduced cortisol variability under heavy training loads (Casanova et al., 2016). Other work suggests that males exhibit stronger acute cortisol responses, whereas females display higher anticipatory tension before exertion (Beckner et al., 2024). However, not all studies report such patterns. Raglin et al. (1991) observed similar psychophysiological responses among male and female swimmers exposed to identical training regimens, indicating that sex-related differences may be context-dependent. Notably, much of the available literature focuses on male athletes (Costello et al., 2014), leaving limited evidence on sex-specific interactions between acute fatigue, mood, and endocrine responses, particularly in rowing, where female participation at the elite level has increased substantially in recent years. Moreover, rowing represents a suitable model for examining sex-specific psychophysiological responses because female and male athletes perform the same standardized competitive task and are exposed to highly comparable training structures and performance demands (Ingham et al., 2002; Cerasola et al., 2020). Maximal rowing exercise also elicits pronounced endocrine, metabolic, and neuromodulatory responses, making it an informative model for investigating acute psychophysiological stress responses in elite athletes [16.

Maximal-effort exercise provides a controlled means of eliciting acute physiological stress, enabling simultaneous assessment of psychological state and biochemical reactivity. Despite extensive research on endocrine responses, mood disturbance, and neurotransmitter dynamics, the relationships between pre-exercise mood and acute hormonal or neuromodulatory responses remain poorly characterized. Addressing this gap requires an integrative psychophysiological approach capable of capturing sex-specific patterns of acute reactivity.

The present study aimed to examine whether pre-exercise mood state was associated with acute hormonal and neuromodulatory responses to a maximal-effort 2000 m rowing test in elite athletes, and whether these responses differed between females and males. We hypothesized that maximal exertion performed after intensified training would induce increases in cortisol and serotonin concentrations together with alterations in dopamine-related responses, and that these responses would differ between female and male athletes. We further hypothesized that pre-exercise mood state would be associated with endocrine and peripheral neurochemical responses during recovery, with sex-specific patterns of association. By integrating mood assessments with acute biochemical responses to maximal exertion, this study was designed to identify sex-specific patterns relevant to fatigue and short-term recovery in high-performance sport.

## Materials and methods

### Ethics approval

The study was approved by the Bioethics Committee of the Nicolaus Copernicus University in Toruń, Collegium Medicum in Bydgoszcz (decision no. KB 312/2024), and conducted in accordance with the Declaration of Helsinki, and retrospectively registered at ClinicalTrials.gov (NCT07243613; 19 November 2025), as an observational study to ensure transparency and compliance with reporting standards. All participants were informed about the study procedures and potential risks and provided written informed consent before participation. Participation was voluntary, and athletes could withdraw from the study at any time without providing a reason.

### Participants

Twelve trained female and sixteen trained male rowers from the Polish Youth National Rowing Team took part in this study. All participants were medically fit and free from any acute or chronic health problems.

All participants completed the same 7-day intensive training camp in the same facility, beginning on the same day (see **Table 1**). Female and male athletes followed identical session schedules and were exposed to the same environmental, nutritional, and recovery conditions. The maximal exertion test was performed on day 8 under identical circumstances for both groups. In national-team practice, such uniform programming is standard, as rowing workloads are prescribed using relative intensities (e.g., %VO_2_max, %HRmax, %power at threshold), while managing it is individualized. This approach ensured standardized external training exposure across all participants, although internal physiological load was not directly monitored during the training camp.

**Table 1:** Training program for the 7 days before the rowing test.

Male and Female	1	2	3	4	5	6	7
Total training time, min/day	110	90	200	105	200	105	100
Time rowed, min/day	65	–	110	95	120	95	40
Distance rowed, km/day	12	–	20	20	22	18	10
Training for force development, min/day	–	–	80	–	–	–	60
Extensive endurance rowing training time, min/day	50	–	110	35	100	95	40
High intensity endurance rowing training time, min/day	15	–	–	60	20	–	–
Unspecific training (running, etc.), min/day	45	90	10	10	60	10	–

Inclusion criteria were: at least five years of rowing training experience, active membership in the national youth team, completion of a standardized 2000-meter ergometer test, and completion of the Profile of Mood States (POMS) questionnaire. Exclusion criteria included the presence of acute or chronic inflammation, pain or injury, use of anti-inflammatory medications, or non-compliance with the study protocol.

All female participants were non-pregnant, non-lactating, and reported regular menstrual cycles (28 ± 4 days). The menstrual cycle phase was not controlled because all measurements were constrained by the fixed schedule of the national-team training camp, which determined the timing of testing and did not allow for individualized adjustments of assessment days. However, this design enabled testing under highly homogeneous training, nutritional, environmental, and recovery conditions during the same preparation period.

Anthropometric measures included body mass, body composition (% fat, fat-free mass) assessed using a bioelectrical impedance analyzer (Tanita BC-418; Tanita Corporation, Tokyo, Japan), and body height measured with a stadiometer (SECA 213; seca GmbH & Co. KG, Hamburg, Germany). Measurements were taken in the morning, after an overnight fast. Performance variables from the 2000-m test (time, mean power, relative power) and blood lactate concentrations before and immediately after the exercise were also recorded. As expected, anthropometric and performance characteristics differed significantly between females and males due to established physiological and morphological sex differences. Descriptive characteristics of study participants are presented in **Table 2**.

**Table 2:** Participant characteristics and 2000-m rowing test results.

Variable	Females (N = 12)	Male (N = 16)
Age (years)	18.75 ± 0.75	19.43 ± 1.09
Body mass (kg)	73.63 ± 3.52	88.74 ± 5.12
Height (m)	176.5 ± 4.23	188.5 ± 5.79
%Fat	18.24 ± 2.33	11.53 ± 2.43
Fat-free Mass (kg)	60.05 ± 4.05	78.46 ± 4.41
Time of exercise (s)	439.83 ± 7.52	380.19 ± 9.29
Power (W)	263.83 ± 13.53	408.87 ± 29.45
Relative power (W·kg^-^¹)	3.59 ± 0.30	4.61 ± 0.33
LA pre-exercise (mmol·L^-^¹)	1.14 ± 0.54	2.70 ± 0.93
LA post-exercise (mmol·L^-^¹)	12.43 ± 2.60	10.95 ± 1.95

Values are mean ± SD. LA: blood lactate.

Since circulating tryptophan levels were analyzed, dietary composition and amino acid intake during the 24 h preceding the exercise test were also recorded. These data are presented in **Table 3**. Dietary intake during the 24 h preceding the exercise test was recorded and analyzed by a certified sports nutritionist using a validated dietary assessment software DietetykPro (DietetykPro Wrocław, Poland), which enabled precise quantification of total energy intake, macronutrients, and amino acid composition, including tryptophan and branched-chain amino acids.

**Table 3:** Dietary composition and amino acid intake 24 h before exercise test.

Variable	Females (24 h)	Males (24 h)	*p-*value
Energy kcal·kg^-^¹	40.56 ± 10.23	40.30 ± 8.17	0.8899
Protein g·kg^-^¹	1.76 ± 0.51	2.09 ± 0.57	0.1843
Carbohydrate g·kg^-^¹	6.46 ± 1.54	5.73 ± 1.39	0.2396
Fat g·kg^-^¹	1.06 ± 0.35	1.06 ± 0.37	0.7091
Tryptophan mg·kg^-^¹	20.76 ± 5.59	24.72 ± 6.35	0.1090
Leucine mg·kg^-^¹	112.90 ± 24.36	139.12 ± 39.03	0.0718
Isoleucine mg·kg^-^¹	70.51 ± 16.88	90.07 ± 21. 10	**0.0259**
Valine mg·kg^-^¹	90.44 ± 23.15	93.75 ± 29.86	0.1154
Tyrosine mg·kg^-^¹	59.33 ± 12.38	64.08 ± 15.73	0.1763
Phenylalanine mg·kg^-^¹	65.39 ± 12.82	78.28 ± 20.56	0.0929

Values are mean ± SD. Bolded values indicate statistically significant differences between groups (*p* < 0.05).

### Study design

The study was conducted during the preparatory phase of the annual training cycle. Athletes first completed anthropometric assessments and the POMS questionnaire, followed immediately by the first blood collection.

Venous blood samples were obtained from the cubital vein at three time points: PRE (collected in the morning between 07:00 and 09:00 to limit circadian influence), POST (within 2 minutes after the 2000-m test), and 3H REC (3 hours post-exercise). Samples were collected into serum tubes with clot activator (Sarstedt AG & Co., Germany), allowed to clot for ~30 min, centrifuged at 3000 rpm for 10 min at 4 °C, aliquoted, and stored at -80 °C until analysis. Following the collection of the PRE blood sample, participants commenced the maximal-effort 2000 m rowing test, which was performed after their usual morning meal, consumed at the training-center dining facility.

The exercise test consisted of a 2000 m maximal-effort trial performed on a Concept2 rowing ergometer (Concept2 Inc., USA). This protocol is widely regarded as the standard laboratory test for assessing rowing performance in elite athletes (Ingham et al., 2002; Cerasola et al., 2020). Before the test, each participant completed a 5-minute individualized warm-up. During the test, participants were verbally encouraged to achieve maximal performance. Moreover, the test outcome contributed to the selection of athletes for championship-level crews; all participants were highly motivated to perform at their maximal capacity. The total time to complete the 2000 m distance and the mean power output were recorded as performance indicators (**Table 2**). All testing procedures were conducted by qualified personnel in compliance with biosafety standards and under identical laboratory conditions to minimize pre-analytical variability.

Serum concentrations of testosterone and cortisol were measured using enzyme-linked immunosorbent assay (ELISA) kits supplied by DiaMetra (Spello, Italy), according to the manufacturer’s instructions. The analytical sensitivity of the assays was 0.10 ng·mL^-^¹ for testosterone and 2.42 ng·mL^-^¹ for cortisol. According to the manufacturer’s specifications, the cortisol assay showed intra-assay and inter-assay coefficients of variation of <8.1% and <6.7%, respectively, while the testosterone assay showed corresponding values of <4.2% and <6.7%.

Serum levels of serotonin, dopamine, GABA, and tryptophan were determined using ELISA kits from SunRed Biotechnology Company (Shanghai, China), following the manufacturer’s protocols. The analytical sensitivity of the assays was as follows: serotonin - 0.388 ng·mL^-^¹, dopamine - 7.043 nmol·L^-^¹, GABA - 1.826 µg·dL^-^¹, and tryptophan - 0.233 ng·mL^-^¹. According to the manufacturer, the ELISA kits for serotonin, dopamine, GABA, and tryptophan showed intra-assay and inter-assay coefficients of variation below 10% and 12%, respectively. All analyses were performed using a SPECTROstar Nano microplate reader (BMG Labtech, Germany).

The testosterone-to-cortisol (T/C) and serotonin-to-dopamine (S/D) ratios were calculated to assess anabolic-catabolic and serotonin-dopamine balance.

Lactate concentration (LA) was assessed in capillary blood samples collected immediately after exercise using the Lactat Photometer and a commercially available diagnostic kit (Diaglobal GmbH, Berlin, Germany), according to the manufacturer’s instructions.

### Psychological assessment

Mood states were assessed using the Profile of Mood States questionnaire in Polish adaptation by Dudek and Koniarek (1987). The POMS is a standardized and widely used instrument designed to evaluate transient mood fluctuations associated with fatigue and recovery processes. It consists of 65 adjectives describing current emotional states, which are rated by participants on a 5-point Likert scale ranging from 0 (“not at all”) to 4 (“extremely”).

The questionnaire yields scores in six subscales: Tension-Anxiety, Depression-Dejection, Anger-Hostility, Vigor-Activity, Fatigue-Inertia, and Confusion-Bewilderment. A Total Mood Disturbance index was also calculated by summing the negative mood dimensions (Tension, Depression, Anger, Fatigue, Confusion) and subtracting the score for Vigor.

Participants completed the POMS questionnaire in the morning, before pre-exercise blood sample collection, following standardized instructions and under the supervision of the research team.

### Statistical analysis

Statistical analyses were performed using Statistica 14.0 (TIBCO Software Inc., Palo Alto, CA, USA) and GraphPad Prism 10.0 (GraphPad Software Inc., San Diego, CA, USA). In accordance with methodological recommendations by Lakens (2022), a sensitivity analysis was performed instead of an *a priori* power analysis because no consistent effect-size estimates are available for acute hormonal and neurotransmitter responses in elite athletes. Sensitivity analysis (G*Power 3.1) indicated that the achieved sample size (females = 12; males = 16) allowed detection of moderate-to-large effects (dz ≈ 0.7-0.8).

The normality of data distribution was assessed using the Shapiro-Wilk test, and the homogeneity of variances was verified with Levene’s test. For all normally distributed variables, a two-way ANOVA (Time × Group) was applied to examine the effects of Time (within-subject factor: PRE, POST, and 3H REC) and Group (between-subject factor: females, males), as well as their interaction (Time × Group). When the assumption of sphericity was violated, the Greenhouse-Geisser correction was applied.

When significant main effects or interactions were observed, Bonferroni-corrected *post hoc* tests were conducted to identify pairwise differences between specific time points and between sexes. In addition, independent samples t-tests (Welch correction) were used for variables measured at a single time point to compare females and males, including dietary variables, and POMS scores. For non-normally distributed data, the Friedman test (within-group) and the Mann-Whitney U test (between-group) were employed.

Exploratory Pearson’s correlations were computed separately for each time point (PRE, POST, and 3H REC) to examine concurrent relationships between mood states and biochemical markers. Given the sample size and the number of explored associations, the correlation analyses were interpreted as exploratory and used to identify biologically plausible psychophysiological relationships that may guide future research. The strength of correlations was interpreted as small (0.10-0.29), moderate (0.30-0.49), or large (≥0.50).

Effect sizes were reported alongside p-values to evaluate the magnitude of observed effects. For ANOVA models, partial eta squared (η²_p_) was calculated and interpreted as small (≥ 0.01), medium (≥ 0.06), or large (≥ 0.14), following conventional criteria (Cohen, 1988; Richardson, 2011). For paired and independent comparisons, effect sizes were calculated as Cohen’s *d* and interpreted as trivial (<0.20), small (0.20-0.49), moderate (0.50-0.79), or large (≥0.80). The strength of Pearson’s correlations was interpreted as small (0.10-0.29), moderate (0.30-0.49), or large (≥0.50), consistent with established conventions for behavioral and sport science research (Cohen, 1988; Hemphill, 2003). Data are expressed as mean ± standard deviation (SD). The level of statistical significance was set at *p* < 0.05.

All participants completed the full testing protocol, and no missing data occurred at any measurement point.

## Results

### Mood states (POMS)

**Figure 1:**
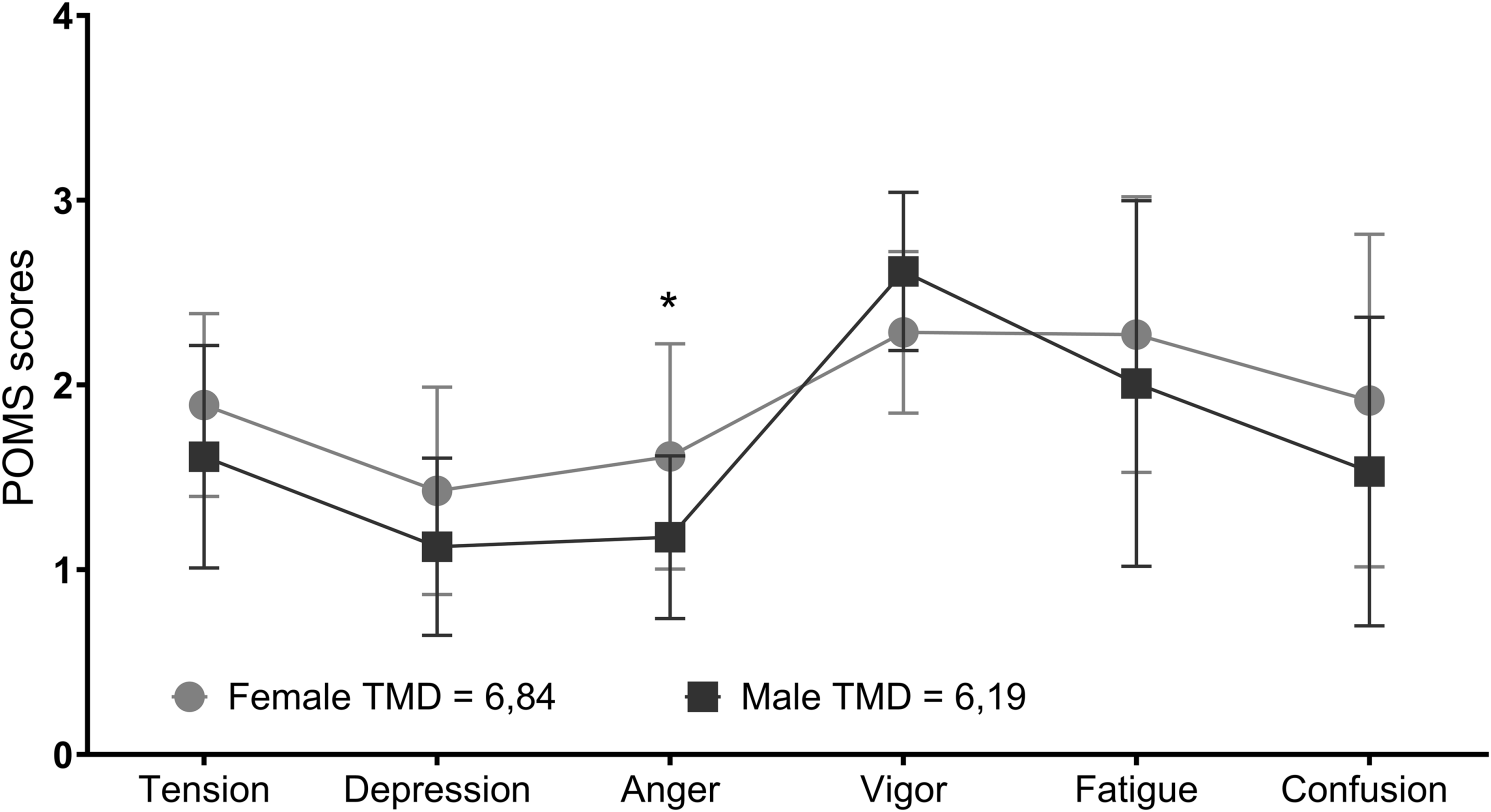
Profile of Mood States in female and male rowers. Values are mean ± SD; TMD - Total Mood Disturbance; * significant difference at *p* < 0.05.

Mood profiles assessed using the Profile of Mood States are presented in **Figure 1**. Both male and female athletes showed a similar distribution of scores across subscales, with the highest values observed for Vigor and the lowest for Depression and Confusion. Female athletes demonstrated slightly higher scores across most negative mood dimensions, resulting in a higher Total Mood Disturbance than male athletes (6.84 vs. 6.19, respectively) however, this difference did not reach statistical significance (*p* = 0.068).

A significant sex difference was observed only in the Anger subscale, with female athletes reporting higher scores *p* = 0.049). No significant sex differences were found for Tension, Depression, Vigor, Fatigue, or Confusion.

### Hormonal responses to exercise

**Figure 2:**
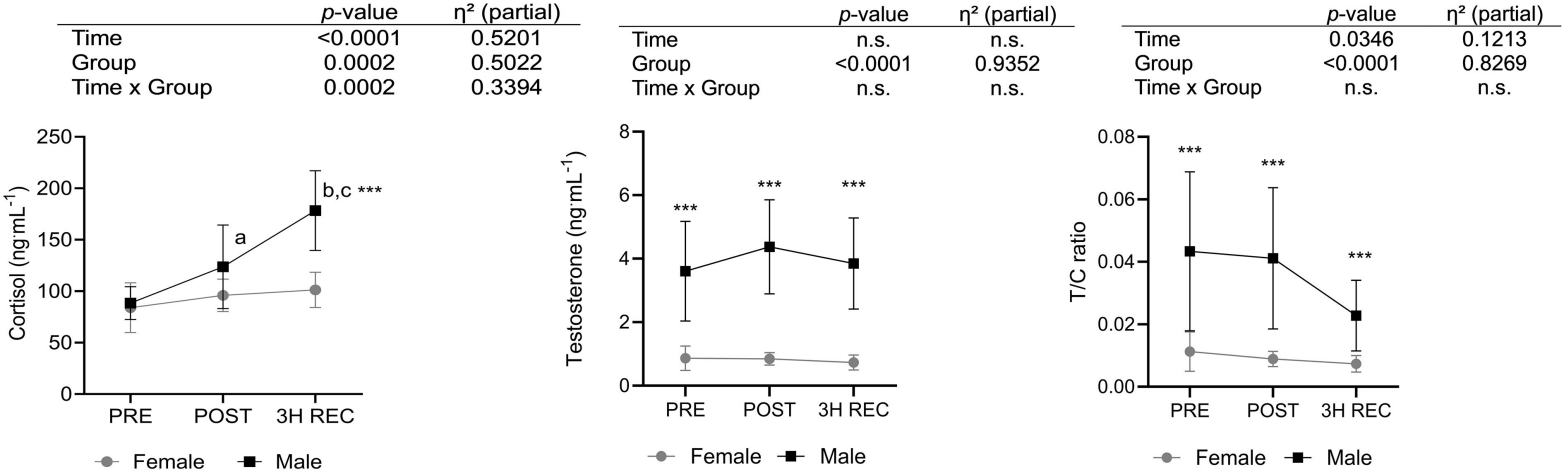
Changes in cortisol, testosterone, and testosterone-to-cortisol ratio (T/C) in male and female participants at PRE, POST, and 3H REC. Data are presented as mean ± SD. PRE - before exercise; POST - immediately after exercise; 3H REC - 3 hours of recovery. ^a^*p* < 0.05 vs. PRE; ^b^*p* < 0.001 vs. PRE; ^c^*p* < 0.001 vs. POST; ***p < 0.001 between groups.

For cortisol, significant main effects of time (*p* < 0.0001, η²_p_ = 0.5201) and group (*p* = 0.0002, η²_p_ = 0.5022), as well as a significant time × group interaction (*p* = 0.0002, η²_p_ = 0.3394), were observed. In males, cortisol increased significantly from PRE to POST and from POST to 3H REC (*d* = 1.14 - 3.04), resulting in a significant overall elevation from PRE to 3H REC. In females, cortisol exhibited non-significant time-dependent changes, with effect sizes indicating small-to-moderate increases (*d* = 0.59 - 0.83). Consequently, cortisol concentrations were significantly higher in males at POST and 3H REC (*p* < 0.001) (**Figure 2**).

For testosterone, only the main effect of group was significant (*p* < 0.0001, η²_p_ = 0.9352), whereas neither time nor the interaction reached significance. Testosterone remained stable across all time points in both sexes (*d* = 0.04 - 0.37). Male athletes consistently showed significantly higher concentrations than female athletes (*p* < 0.001), reflecting expected physiological sex differences (**Figure 2**). This very large group effect reflects the expected physiological difference in circulating testosterone concentrations between female and male athletes.

For the T/C ratio, significant main effects of time (*p* = 0.0346, η²_p_ = 0.1213) and group (*p* < 0.0001, η²_p_ = 0.8269) were found, with no significant interaction. Both groups demonstrated non-significant decreases across time (*d* = 0.05 - 0.72). At all time points, males exhibited significantly higher T/C values than females (*p* < 0.001), consistent with physiological differences in anabolic-catabolic balance (**Figure 2**).

### Neurotransmitter responses

**Figure 3:**
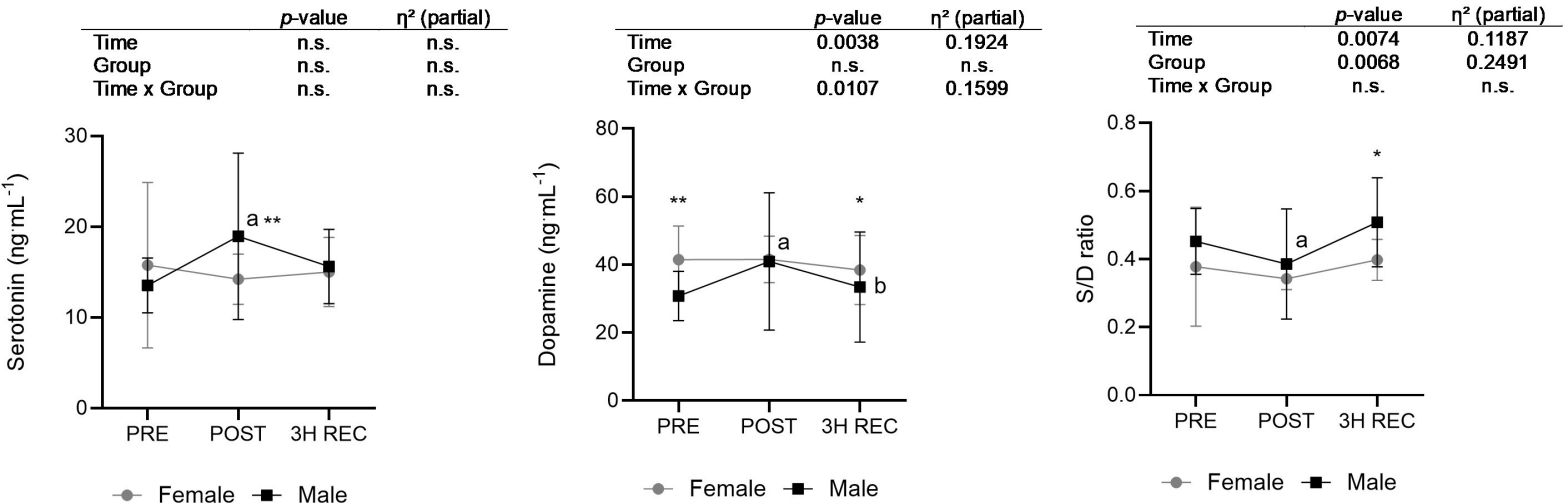
Changes in serotonin, dopamine, and serotonin-to-dopamine ratio (S/D) in male and female athletes at PRE, POST, and 3H REC. Values are mean ± SD. PRE - before exercise; POST - immediately after exercise; 3H REC - 3 hours of recovery. ^a^*p* < 0.05 vs. PRE; ^b^*p* < 0.05 vs. POST; *p < 0.05, **p < 0.01 between groups.

For serotonin, no significant main effects of time, group, or time × group interaction were detected (*p* > 0.05). Although no significant overall ANOVA effects were observed for serotonin, exploratory *post hoc* comparisons indicated a significant PRE-to-POST increase in males (d = 0.79), followed by a return toward baseline at 3H REC (d = 0.47–0.58), whereas females showed only small, non-significant fluctuations across time (d = 0.11–0.24). Serotonin concentrations were significantly higher in female athletes at POST (*p* = 0.045), with no significant between-group differences at PRE or 3H REC (**Figure 3**).

For dopamine, significant main effects of time (*p* = 0.0038, η²_p_ = 0.1924) and a significant time × group interaction (*p* = 0.0107, η²_p_ = 0.1599) were observed, while the main effect of group was not significant. In males, dopamine increased significantly from PRE to POST and decreased significantly from POST to 3H REC (*d* = 1.07 - 1.38), resulting in a minimal PRE to 3H REC difference (*d* = 0.21). In females, dopamine remained stable across time, with small, non-significant effect sizes (*d* = 0.01-0.36). Between-group comparisons showed higher dopamine in females at PRE (*p* < 0.01) and higher concentrations in males at 3H REC (*p* < 0.05), with no significant difference at POST (**Figure 3**).

For the S/D ratio, significant main effects of time (*p* = 0.0074, η²_p_ = 0.1187) and group (*p* = 0.0068, η²_p_ = 0.2491) were identified, whereas the interaction was not significant. In males, the S/D ratio decreased significantly from PRE to POST and showed a non-significant increase at 3H REC (*d* = 0.49 - 0.84). In females, the S/D ratio displayed non-significant time-dependent fluctuations (*d* = 0.16 - 1.16). At 3H REC, S/D values were significantly higher in males (*p* < 0.05), with no significant between-group differences at PRE or POST (**Figure 3**).

### GABA and tryptophan

**Figure 4:**
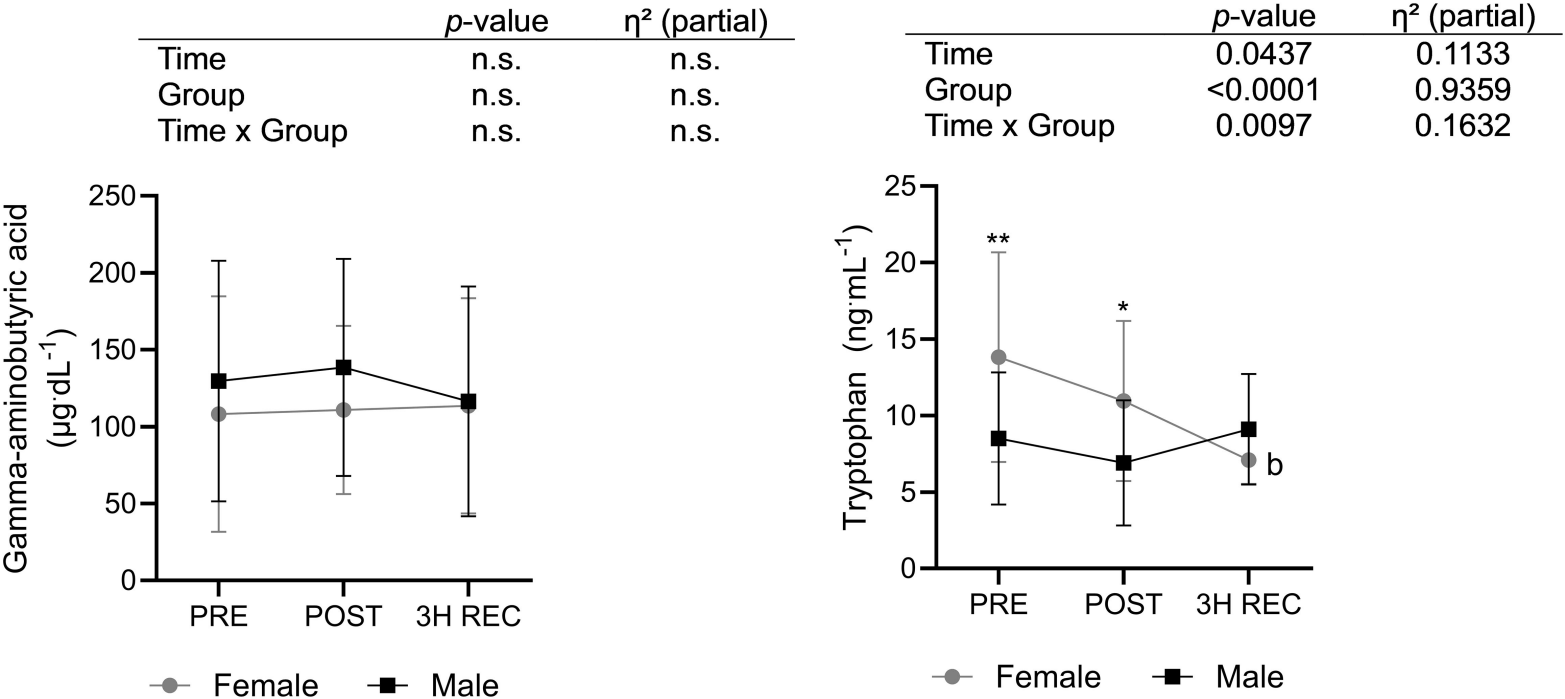
Changes in gamma-aminobutyric acid (GABA) and tryptophan concentrations in male and female athletes at PRE, POST, and 3H REC. Values are mean ± SD. PRE - before exercise; POST - immediately after exercise; 3H REC - 3 hours of recovery. *p < 0.05, **p < 0.01 between groups; ^b^*p* < 0.05 vs. POST.

For GABA, no significant main effects of time, group, or time × group interaction were observed (all *p* > 0.05). In males, GABA showed small, non-significant fluctuations across measurements, with effect sizes indicating minimal PRE-to-POST, PRE-to-3H REC, and POST-to-3H REC differences (*d* = 0.12 - 0.30). In females, GABA also remained stable over time, with very small effect sizes (*d* = 0.04 - 0.07) and no statistically significant within-group changes. No significant between-group differences were detected at PRE, POST, or 3H REC (**Figure 4**).

For tryptophan, significant main effects of time (*p* = 0.0437, η²_p_ = 0.1133) and group (*p* < 0.0001, η²_p_ = 0.9359) were identified, as well as a significant time × group interaction (*p* < 0.0097, η²_p_ = 0.1632) were identified. In females, tryptophan decreased progressively across the three time points, with a significant reduction from PRE to 3H REC (*p* < 0.05) and effect sizes indicating moderate to large changes (*d* = 0.47 - 1.35). In males, tryptophan values decreased from PRE to POST and showed a slight increase at 3H REC, but none of these changes reached statistical significance, with small-to-moderate effect sizes (*d* = 0.15 - 0.57). Between-group comparisons demonstrated higher tryptophan concentrations in females at PRE (*p* < 0.01) and POST (*p* < 0.05), while no significant difference was noted at 3H REC (**Figure 4**). The very large group effect should be interpreted in the context of marked baseline differences in circulating tryptophan concentrations between female and male athletes.

### Correlations between biochemical markers and mood

Exploratory correlation analyses were conducted to examine associations between pre-exercise mood dimensions and biochemical markers at each time point (**Table 4**).

**Table 4:** Correlations between biochemical markers and mood states (POMS scores) in male and female athletes.

Timepoint	Biomarker	POMS	Females *(r [95% CI])*	*P* value	Males *(r [95% CI])*	*P* value
3HRecovery	Cortisol	Anger	-0.66 [-0.85, -0.21]	0.019	–	–
3HRecovery	Cortisol	Depression	-0.64 [-0.84, -0.18]	0.026	–	–
3HRecovery	Cortisol	Tension	-0.65 [-0.83, -0.22]	0.021	–	–
3HRecovery	Cortisol	POMS TMD	-0.63 [-0.82, -0.19]	0.030	–	–
Post	Tryptophan	Depression	0.66 [0.22, 0.86]	0.021	-0.60 [-0.82, -0.13]	0.014
Post	Serotonin	Depression	–	–	0.52 [0.07, 0.78]	0.038
Post	Dopamine	Vigor	–	–	-0.56 [-0.79, -0.10]	0.026
Pre	Cortisol	Vigor	–	–	-0.50 [-0.77, -0.02]	0.046

Values represent Pearson’s correlation coefficients (r) with 95% confidence intervals (CI). Significant correlations (*p* < 0.05) are shown. POMS - Profile of Mood States; TMD - total mood disturbance; PRE - before exercise; POST - immediately after exercise; 3H REC - 3 hours of recovery.

In female athletes, cortisol concentrations measured during recovery were negatively correlated with Anger, Depression, Tension, and Total Mood Disturbance, suggesting that lower mood disturbance scores were associated with higher cortisol levels at 3H REC. Additionally, post-exercise tryptophan concentration showed a positive correlation with Depression.

In male athletes, post-exercise tryptophan concentration correlated negatively with Depression, whereas serotonin at POST correlated positively with the same dimension. Dopamine concentrations at POST were negatively associated with Vigor, and pre-exercise cortisol concentrations correlated negatively with Vigor. **Table 4** summarizes significant correlations (*p* < 0.05) along with 95% confidence intervals for male and female athletes.

## Discussion

The present study provides an integrated analysis of acute endocrine and neuromodulatory responses to maximal exertion in elite female and male athletes, demonstrating that pre-exercise mood state is differentially associated with hormonal and neurotransmitter dynamics across sex. The findings show that although both groups completed a standardized maximal 2000-m effort, the underlying physiological responses and their psychological correlates showed distinct response patterns. Our results highlight that biological sex may influence the coordination between psychological state and acute physiological stress responses, underscoring the need to consider sex-specific regulatory mechanisms when interpreting biochemical markers in elite athletes.

The endocrine responses observed in this study were broadly consistent with established models of acute HPA-axis activation during high-intensity exercise, although their magnitude differed between females and males. Cortisol reactivity is known to vary according to biological sex, training load, and emotional state (Di Corrado et al., 2014; Ostapiuk-Karolczuk et al., 2025a). In line with previous evidence males showed a clearer cortisol increase across time points, whereas females exhibited only small changes, reflect sex-related differences in stress responsiveness under accumulated training load (Hooper and Mackinnon, 1995; Di Corrado et al., 2014; Beckner et al., 2024). The cortisol response observed in both groups, even after seven days of intensified training, may reflect physiological responses to short-term accumulated training stress, in accordance with previous findings in rowers, combat athletes, and team sport players (Ingham et al., 2002; Costello et al., 2014; Ficarra et al., 2024). At the same time, this interpretation should remain cautious, as the present study did not include longitudinal performance monitoring or a more comprehensive assessment of recovery status.

The decline in the testosterone-to-cortisol (T/C) ratio reflected an acute catabolic shift induced by maximal exertion and intensified training load. Comparable reductions in the T/C ratio were observed in females and males, with no significant interaction, suggesting a broadly similar proportional endocrine response despite marked sex-related differences in hormone concentrations. Reduced T/C ratios have long been associated with training strain and fatigue accumulation (Meeusen et al., 2013), especially when combined with suppressed cortisol reactivity, reduced anabolic function, or persistent mood disturbance. However, interpretation of the T/C ratio requires context, particularly in relation to biological sex and training demands (Thiel et al., 2011; Cadegiani and Kater, 2017). Importantly, emerging findings indicate that sex moderates the interpretation of T/C ratios; for example, Ficarra et al. 8] reported that elite female rowers with higher T/C ratios performed worse, highlighting the need to avoid uniform thresholds when interpreting endocrine markers.

Peripheral neurochemical responses showed patterns potentially associated with fatigue-related psychophysiological processes, including increased serotonin, reduced dopamine, and an elevated serotonin-to-dopamine ratio. This pattern may be discussed in the context of fatigue-related monoaminergic models. Previous studies have shown that increased serotonergic activity, together with reduced dopaminergic tone, has been linked to fatigue-related processes, heightened perceived exertion, and reduced motivational drive during strenuous exercise (Davis et al., 2000; Heijnen et al., 2016; Zimmer et al., 2016; Cordeiro et al., 2017; Kavanagh et al., 2019). In the present study, these monoaminergic dynamics appeared to be sex-specific: dopamine increased markedly in males at POST and decreased during recovery, whereas females showed only trivial fluctuations, suggesting that peripheral dopamine-related responses may show greater exercise-induced modulation in males during acute fatigue and early recovery. (**Figure 3**). Although such patterns are typically described in the context of accumulated exertion, the present findings suggest that peripheral monoamine-related responses may also change after a single maximal bout performed in a competition-like context.

The observed changes in tryptophan and GABA provide additional information on the neurochemical pathways involved in acute regulatory responses to maximal exertion. Tryptophan availability is a major determinant of serotonin synthesis during exercise 39]. Our results showed a moderate-to-large decrease in circulating tryptophan exclusively in females, whereas males exhibited only small changes, indicating a sex-specific regulation of tryptophan availability during maximal exertion (**Figure 4**). Because tryptophan availability may influence serotonin synthesis during exercise (Fernstrom and Fernstrom, 2006), these distinct responses may contribute to the pronounced sex differences observed in serotonergic activity. Importantly, dietary tryptophan intake was comparable between females and males, while isoleucine was the only analyzed amino acid that differed significantly between sexes. (**Table 3**), indicating that the marked post-exercise reduction in tryptophan in females reflects physiological rather than nutritional mechanisms, potentially involving sex-specific differences in albumin binding and displacement of tryptophan by free fatty acids (Badawy, 2015), greater lipid mobilization during exercise (Tarnopolsky, 2008), and sex-dependent variation in exercise-induced proteolysis (Devries and Phillips, 2015). Notably, the fact that women exhibited a faster and more pronounced post-exercise decline underscores a biologically meaningful female sensitivity to metabolic stress.

Previous literature indicates that acute exercise may be accompanied by changes in peripheral GABA concentrations, which have been discussed in relation to exercise-related neurochemical and endocrine regulation (Powers et al., 2008; Lyssikatos et al., 2023b). However, in our study, GABA responses did not differ between sexes and remained relatively stable across time points, serum GABA may be less responsive to acute maximal exertion than monoaminergic markers. In contrast to the dynamic serotonergic response, the stability of serum GABA may suggests that peripheral GABAergic markers may be less sensitive to acute maximal exertion and early recovery. This leaves the systemic and central mechanisms involved in post-exercise down-regulation open for further investigation.

A novel contribution of this study is the integration of the immediate pre-exercise psychological state with acute hormonal and neurochemical outcomes (**Figure 1**, **Table 3**). Although both groups showed typical mood profiles for elite athletes, several sex-specific psychophysiological relationships emerged. In females, higher levels of anger, tension, depression, and overall mood disturbance were associated with lower cortisol concentrations during recovery. This is noteworthy because cortisol changed only slightly in females, yet clear correlations were present, which may reflect an association between emotional state and aspects of HPA-axis recovery even under conditions of minimal hormonal change. This pattern may be interpreted in the context of attenuated HPA-axis reactivity described in female athletes experiencing elevated emotional strain or periods of heavy training, including flattened cortisol rhythms, reduced responsiveness, and altered stress perception (Hooper and Mackinnon, 1995; Di Corrado et al., 2014). Taken together, these exploratory findings may suggest that in females, baseline emotional state could be associated with aspects of endocrine recovery following maximal exertion, potentially reflecting sex-specific patterns of HPA regulation or greater integration of affective and endocrine pathways.

In contrast, male athletes exhibited a distinct psychophysiological profile. Lower vigor and higher depressive symptoms correlated with higher serotonin and lower dopamine after exercise. These exploratory associations appeared to align with the observed male biochemical response profile, in which dopamine showed large POST-to-3H REC changes, and serotonin increased proportionally more than in females, supporting the role of peripheral monoamine-related responses in fatigue-related processes in males. This association reflects monoaminergic patterns previously described in fatigued endurance athletes and combat sport competitors, where dopaminergic withdrawal and serotonergic dominance have been linked to reduced motivation, diminished drive, fatigue-related responses, and impaired performance regulation (Meeusen, 2006; Slimani et al., 2018). Similar profiles have been reported in athletes undergoing intense competition or psychological stress, where more negative mood states coincide with neurochemical signatures suggestive of greater psychophysiological strain (Heijnen et al., 2016; Seddik et al., 2025). Our findings extend this evidence by demonstrating that these relationships may be sex-specific and become apparent even in a single, standardized maximal-effort test.

The exploratory correlations showed clear sex-specific patterns. In females, the consistent association between negative mood dimensions (anger, tension, depression, total mood disturbance) and lower cortisol concentrations during recovery reinforces the concept that females may exhibit a dampened HPA-axis response in the presence of psychological strain. The present results therefore may suggest that, in females, endocrine recovery after maximal exertion may be particularly closely associated with affective state, despite relatively small absolute changes in cortisol. This resembles findings in overloaded female athletes showing blunted cortisol rhythms, attenuated HPA reactivity, and heightened emotional strain (Hooper and Mackinnon, 1995; Di Corrado et al., 2014). Such relationships may suggest that hormonal responsiveness to maximal metabolic load in females may be particularly closely associated with pre-exercise emotional state, reflecting potential differences in stress appraisal, coping strategies, or neuroendocrine integration.

In males, the exploratory association between mood and monoaminergic responses, specifically reduced vigor and higher depressive symptoms associated with suppressed dopamine and elevated serotonin, mirrors neurochemical patterns previously described in models of fatigue-related psychophysiological responses. These models emphasize that a serotonergic shift, paired with reduced dopaminergic activity may contribute to greater perceived exertion, reduced motivational drive, and earlier task disengagement (Meeusen, 2006; Meeusen and Roelands, 2018). Similar neurochemical-affective coupling has been noted in combat sport athletes and endurance competitors under highly competitive stress (Slimani et al., 2018)further supporting the notion that these relationships reflect meaningful psychophysiological mechanisms rather than isolated observations. Our results may suggest that in males, psychological state may be associated with acute post-exercise recovery mainly through monoaminergic pathways.

Together, these sex-specific associations, HPA-linked in females and monoamine-linked in males, have not been widely documented in elite athletes or in studies integrating mood, hormones, and neurotransmitters within a unified approach (van Paridon et al., 2017). They may indicate potential sex-related differences in psychophysiological associations that require confirmation in larger studies. In females, mood disturbance was more closely related to hormonal recovery dynamics, whereas in males it aligned more strongly with neurotransmitter responses. Considering the distinct cortisol, dopamine, and tryptophan responses documented in this study, our findings may suggest that the biological mechanisms underlying fatigue differ substantially between sexes, even under identical performance demands. Considering these factors together improves the understanding of how fatigue develops and resolves and addresses an important gap in sports science, where sex differences are often noted but rarely examined jointly across psychological, endocrine, and neurochemical systems. Given the limited sample size and exploratory nature of these analyses, these associations should be interpreted cautiously and considered hypothesis-generating rather than confirmatory.

From a practical perspective, these findings highlight the value of individualized monitoring in elite sport. Regular assessment of mood, for example through the POMS questionnaire, may help identify early signs of psychological strain, particularly in female athletes, who appeared more sensitive to training load in this study. Peripheral neurochemical indicators such as the serotonin-dopamine balance or tryptophan levels may be especially informative in male athletes (Yamashita and Yamamoto, 2017; Yang et al., 2024), while endocrine markers like the T/C ratio should be interpreted with attention to sex-specific differences (Ficarra et al., 2024; Mondal et al., 2025). Together, these results suggest that tailored monitoring strategies, emphasizing endocrine markers in females and monoaminergic markers in males, may improve early detection of fatigue and guide individualized recovery planning. The hormonal and neurochemical responses observed in the present study suggest that elite athletes exhibited measurable acute physiological and psychophysiological responses after a short period of intensified training. Nevertheless, the present findings are not sufficient to determine whether these responses reflected functional overreaching or normal acute adaptation to accumulated training load.

In summary, this study shows that maximal exertion performed after an intensified training period was accompanied by coordinated endocrine and peripheral neurochemical responses associated with acute physiological and psychophysiological stress, while also revealing sex-specific links between mood, hormonal regulation, and peripheral neurotransmitter response. By examining these systems together, under sport-specific high-load conditions, this work addresses a notable gap in the literature and provides novel evidence that psychological state can shape physiological stress responses in a sex-dependent manner. These findings offer a more integrated understanding of fatigue regulation in elite athletes and emphasize the importance of including sex-specific psychophysiological markers in monitoring and training strategies.

## Conclusions

The present study suggests that the relationship between pre-exercise mood state and acute physiological responses to maximal exertion may differ between female and male elite rowers, despite identical training and testing conditions. These findings support the view that biological sex should be considered when interpreting psychophysiological responses to exercise and when evaluating short-term fatigue and recovery processes in high-performance sport.

More broadly, the results highlight the potential value of combining psychological assessment with biochemical monitoring to obtain a more integrated understanding of athlete readiness and stress reactivity. Given the exploratory nature of the correlation analyses and the limited sample size, these observations should be interpreted with appropriate caution.

### Study limitations

This study has several limitations that should be considered when interpreting the findings. The menstrual cycle phase of female athletes was not controlled because the testing schedule followed the fixed organization of the national team training camp. Therefore, possible intra-female hormonal variability should be considered when interpreting the sex-specific responses. However, this setting also provided highly homogeneous training, nutritional, environmental, and recovery conditions, which is rare in elite sport research. Although all participants lived, trained, and ate under the same conditions, factors such as sleep quality, psychological stress external to training, and individual recovery strategies were not monitored and may have contributed to variability in physiological responses. The sample size reflects the restricted availability of elite athletes and limits the statistical power of exploratory associations, particularly sex-specific correlation analyses; therefore, these associations should be considered hypothesis-generating until confirmed in larger cohorts. In addition, because multiple exploratory correlations were examined, the possibility of Type I error should be considered when interpreting these findings. Finally, the findings pertain to a highly trained national-level rowing cohort, which may limit generalizability to other athletic populations or to recreationally active individuals. Neurotransmitter-related markers were assessed in peripheral serum samples and therefore should not be interpreted as direct measures of central nervous system activity.

### Practical implications

Present findings indicate that monitoring strategies in elite athletes should take biological sex into account. In female athletes, pre-exercise mood showed a strong association with cortisol levels during recovery, despite only small absolute hormonal changes. This may suggest that affective state is associated with endocrine responses during recovery, particularly anger, tension, and depressive symptoms. Psychological factors may therefore represent an important consideration when interpreting recovery-related responses in female athletes during periods of intensified training., Because tryptophan decreased only in females, nutritional approaches aimed at maintaining stable tryptophan availability may warrant further investigation in the context of recovery and fatigue-related processes. In male athletes, mood was closely linked to dopaminergic and serotonergic responses. Lower vigor and higher depressive symptoms were associated with reduced dopamine and increased serotonin after maximal exertion, suggesting sex-specific associations between psychological state and peripheral monoamine-related responses. Monitoring motivational state and regulating cumulative sympathetic load may therefore be relevant considerations in athlete recovery management. Overall, endocrine indicators and peripheral monoamine-related markers may show different patterns of association with psychological state in female and male athletes. However, these observations require confirmation in larger cohorts before being translated into sex-specific monitoring strategies.

## Data Availability

The raw data supporting the conclusions of this article will be made available by the authors, without undue reservation.
